# Challenges in Conducting Exercise Recovery Studies in Older Adults and Considerations for Future Research: Findings from a Nutritional Intervention Study

**DOI:** 10.3390/geriatrics9050116

**Published:** 2024-09-10

**Authors:** Eleanor Jayne Hayes, Christopher Hurst, Antoneta Granic, Avan A. Sayer, Emma Stevenson

**Affiliations:** 1 Health and Life Sciences, Northumbria University, Newcastle upon Tyne NE1 8ST, UK; 2 AGE Research Group, Faculty of Medical Sciences, Translational and Clinical Research Institute, Newcastle University, Newcastle upon Tyne NE4 5PL, UK; christopher.hurst@newcastle.ac.uk (C.H.); antoneta.granic@newcastle.ac.uk (A.G.); avan.sayer@newcastle.ac.uk (A.A.S.); 3 NIHR Newcastle Biomedical Research Centre, Newcastle upon Tyne Hospitals NHS Foundation Trust, Cumbria, Northumberland, Tyne and Wear NHS Foundation Trust and Newcastle University, Newcastle upon Tyne NE4 5PL, UK; 4 School of Biomedical, Nutritional and Sport Sciences, Newcastle University, Newcastle upon Tyne NE2 4HH, UK; emma.stevenson@newcastle.ac.uk

**Keywords:** resistance exercise, older adults, exercise-induced muscle damage, methodology

## Abstract

Maximising the potential benefit of resistance exercise (RE) programs by ensuring optimal recovery is an important aim of exercise prescription. Despite this, research surrounding recovery from RE in older adults is limited and inconsistent. The following randomised controlled trial was designed to investigate the efficacy of milk consumption for improving recovery from RE in older adults. However, the study encountered various challenges that may be applicable to similar studies. These include recruitment issues, a lack of measurable perturbations in muscle function following RE, and potential learning effects amongst participants. Various considerations for exercise research have arisen from the data which could inform the design of future studies in this area. These include (i) recruitment—consider ways in which the study design could be altered to aid recruitment or allow a longer recruitment period; (ii) learning effects and familiarisation—consider potential learning effects of outcome measures and adjust familiarisation accordingly; (iii) identify, validate and optimise protocols for outcome measures that are applicable for the specific population; (iv) adjust the exercise protocol according to the specific aims of the study (e.g., are you replicating a usual exercise bout or is the intent to cause large amounts of muscle damage?).

## 1. Introduction

Maximising the potential benefit of resistance exercise (RE) programmes by ensuring optimal recovery is an important aim of exercise prescription [1]. This includes minimising the magnitude of exercise-induced muscle damage and reducing the time for muscle function and soreness to return to baseline values. For older adults, this may be particularly important as any transient decrements in physical functioning resulting from RE could be detrimental when performing habitual daily activities (e.g., walking, climbing stairs, and household chores) or could increase falls risk [2,3]. Likewise, poor or delayed recovery resulting in prolonged muscle soreness may affect motivation for continued engagement in RE programmes [1].

Despite the increasing interest in RE for healthy ageing, a systematic scoping review of recovery from RE in older adults found the literature to be limited and inconsistent [4,5]. Specifically, our recent review noted variability in study protocols, inconsistency of reported outcomes for physical functioning, inconsistencies in the magnitude of exercise-induced muscle damage and time to recovery, and a paucity of intervention studies. We also found that outcome measures applicable to daily living in older adults such as timed up-and-go (TUG), time to five chair rises, and postural stability were not widely reported [4]. Notably, the review could identify no nutritional intervention studies for recovery from resistance exercise in older adults [4].

Optimising recovery is important for limiting muscle soreness and restoring muscle strength following resistance exercise. Nutritional interventions, particularly those high in protein, are widely used in younger adults and athletes for exercise recovery [6,7]. A protein-rich whole food that has gained popularity as an exercise recovery aid is cow’s milk, which can stimulate muscle protein synthesis to similar levels as whey, alongside having a favourable macro- and micronutrient content that could contribute to exercise recovery [8,9]. For older adults, milk has already been reported to be acceptable [10,11,12], whilst having minimal effects on appetite and satiety [13]—an important consideration for a population at high risk for low energy intake [14].

The following randomised controlled trial was designed to investigate the efficacy of milk consumption for improving recovery from RE in older adults. It was hypothesized that consuming two servings of 500 mL of whole milk would decrease the magnitude of exercise-induced muscle damage and improve recovery time compared to the same volume of skimmed milk and a control drink. Due to challenges with recruitment, statistical power for detecting between-group differences was not achieved; hence, all results should be interpreted with caution, and no conclusions should be drawn as to the effectiveness of whole milk as an exercise recovery supplement. Despite this, this study yielded important findings regarding methodological considerations for conducting similar studies. This article will describe the study methodology and then discuss how these considerations have arisen from our data and how they could be considered in the design and delivery of future research in the area.

## 2. Materials and Methods

### 2.1. Participants

Participants were recruited from Newcastle upon Tyne and surrounding areas between March and September 2022. The primary method for recruitment was via VOICE^®^ (https://voice-global.org/), a public involvement platform that mails opportunities to members of the community who are interested in being involved with health research. The study was also advertised via an online university staff bulletin, social media (Facebook^®^, Twitter^®^), and posters were displayed at various locations (e.g., public libraries) across Newcastle upon Tyne. Other community groups were emailed with information, and posters and were asked to circulate these to their members. All interested individuals were sent a participant information pack via email or post, describing the study requirements and procedures before deciding if they wished to participate.

Participants were required to be over 70 years of age, willing and able to drink bovine milk, have no medical contraindications to RE (assessed by completion of a Physical Activity Readiness Questionnaire (PARQ+) [15]), and report that they did not currently, or have not within the last year, performed structured RE. The latter requirement was included to minimize the repeated bout effect, defined as “the adaptation whereby a single bout of eccentric exercise protects against muscle damage from subsequent eccentric bouts” [16]. Using data from Rankin et al. (2018) [17] describing changes in peak torque of the knee flexors at 60°/s between baseline and 24 h for a milk versus carbohydrate drink, a power analysis using G*power (version 3.1.9.2) with ANOVA repeated measures, within and between interactions (groups = 3, assessment times = 5, and correlation among repeated measures = 0.5) was performed to determine appropriate sample sizes. With an effect size of 0.22, a 2-tailed significance level (α) of 0.05, and the desired power (1-β) of 0.80, a sample size of 36 with 12 participants in each group was needed.

### 2.2. Experimental Design

In a single-blind, placebo-controlled, parallel groups design, participants were randomized to one of three experimental treatment arms: skimmed milk, whole milk, or isocaloric red grape juice. Participants were randomly stratified based on sex and maximal isometric voluntary contraction (MIVC) of the knee extensor as blocking factors using co-variate adaptive randomization [18]. MIVC stratification thresholds were ≤100 Nm, 100–150 Nm, and >150 Nm. These data were collected from the screening and familiarisation visit conducted >2 weeks before experimental testing.

**Figure 1:**
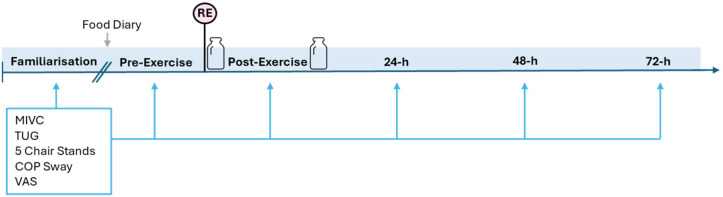
Protocol schematic. MIVC: maximal isometric voluntary contraction, TUG: timed up-and-go, COP: centre of pressure, VAS: visual analogue scale, RE: resistance exercise, h: hours.

Participants were asked to report to the university laboratory on five separate occasions. The first visit to the laboratory included health screening (including PARQ+ [15]), familiarisation of function tests and determination of maximal strength (one-repetition maximum; 1-RM) values. Experimental testing was then conducted over four consecutive visits to assess recovery up to 72 h post-exercise. Participants reported to the laboratory at the same time each day (±1 h). Upon arrival to the first experimental visit, baseline measures of MIVC, perceived recovery, muscle soreness, and physical functioning were conducted. The participant then performed an RE protocol (described below). Further measures of maximal isometric strength, perceived recovery, muscle soreness, and physical functioning were repeated immediately after, and 24, 48, and 72 h after the resistance exercise protocol (Figure 1).

### 2.3. Pre-Laboratory Stipulations

Participants were asked to avoid bouts of strenuous physical activity 24 h prior to the first experimental visit, and for the remainder of the trial (96 h in total). Participants were also instructed to avoid consuming any nutritional supplement (e.g., whey protein, vitamins, creatine), or receive any alternative exercise recovery treatments (e.g., massage, cold water immersion, hot baths, etc.) for the duration of the study. Participants were given a paper template to record their food and drink intake in a weighed food diary for the 24 h prior to the first experimental visit. This was analysed for total energy and macronutrient intake to ensure that diet was not significantly different between groups at baseline.

### 2.4. Resistance Exercise Protocol

In an attempt to replicate a pragmatic RE session to ensure ecological validity, the participants performed four sets of ten repetitions at 70% 1-RM of three lower limb exercises: leg press, knee extensions, and hamstring curls. These were performed on fixed weights machines (Attack Fitness, Stoke-on-Trent, UK/XS Sports, Shrewsbury, UK), with two minutes of rest between each set. Participants were instructed to perform the eccentric components of the lift in a controlled manner. This is similar to the exercise protocol recommended by a meta-analysis seeking the most effective RE protocol for older adults [19], and to the protocol used by the singular recovery intervention study in older adults [20], but used larger muscle groups of the lower limbs and was altered after pilot testing to reflect what researchers thought was attainable using our specific equipment. After each exercise, participants were asked to provide a rating of perceived exertion (RPE) using the Borg (6–20) scale [21,22], to enable comparison of effort between groups.

### 2.5. Estimating 1-RM

During the screening visit, we estimated 1-RM for each of the exercises using Brzycki’s method for predicting a 1-RM from repetitions to fatigue [23]. This method has been shown to be valid for use in older adults [24]. This protocol was followed due to the potential risks and practical implications, such as being unfamiliar with maximal lifting, that arise from older adults attempting a 1-RM lift. This protocol uses sub-maximal efforts to estimate maximal strength.

### 2.6. Nutrition Intervention

Immediately after performing the RE protocol, participants consumed a 500 mL bolus intake of either (i) whole milk, (ii) skimmed milk, or (iii) isocaloric control drink. The isocaloric control drink was red grape juice and was chosen to match whole milk energy content (Table 1). Participants were provided with an additional 500 mL of each supplement to consume within three hours of leaving the laboratory. This volume (500 mL) was given as it provides approximately 20 g of protein needed to stimulate muscle protein synthesis above that provided by resistance exercise [25,26], is routinely used in similar studies in younger adults [9,27], and has previously been used in older adults [12]. Compliance was assessed by asking for verbal confirmation at their next study visit that they had consumed the supplement within the required time.

### 2.7. Maximal Isometric Voluntary Contraction

Maximal isometric voluntary contraction (MIVC) of the knee extensors was measured during familiarisation and during all experimental visits (Biodex Isokinetic Dynamometer System 4 ProTM & Advantage BX Software 5.3X, Biodex Medical Systems Inc., New York, NY, USA). Participants were seated upright, with the attachment arm strapped to the distal part of the lower leg, just above the ankle joint, and at a knee flexion of 90°. To prevent any abduction, adduction, or flexion of the hip, the distal part of the thigh was strapped to the chair, and further straps were applied to the waist and torso. Participants were positioned so that the patellofemoral joint was aligned with the pivot point of the attachment arm. The configuration of the dynamometer was recorded to be replicated during each visit. During data collection, participants were asked to place their arms over their chest, rather than gripping the available handles, to further isolate the knee extensors.

Participants performed four unilateral maximal isometric contractions, separated by 30 s of rest. After a three-second countdown, participants were instructed to maximally extend their knee flexor after hearing the command “push” and hold the contraction for five seconds. Participants received no verbal encouragement during contraction, only a countdown of the five seconds. During all experimental testing, MIVC was measured using the dominant leg only, defined as the leg that achieved the highest peak torque during familiarisation. This method is well used within exercise recovery studies [4].

### 2.8. Muscle Soreness

Participants were asked to rate their passive perceived muscle soreness using a 100 mm visual analogue scale, with 0 representing “no pain” and 100 indicating “extreme pain”. Pain was indicated by drawing a vertical line on the scale. Line placement was then measured with a ruler from 0 and recorded in millimetres (mm). The use of visual analogue scales is common within the exercise recovery literature and has previously been used with older adults [4].

### 2.9. Postural Stability

Currently, only one study has investigated the effects of resistance exercise on postural control in the 72 h following exercise in older adults [4,20]. Using a similar methodology to the previous study [20], postural stability was quantified by the assessment of thcentre of pressure (COP) sway. Quiet stance COP sway was measured three times for 30 s at each time point. The mean of the three trials was calculated and used for statistical analysis. Participants stood on a Portable Force Platform (PASPORT Force Platform PS-2141, PASCO, Roseville, CA, USA), with their feet shoulder width apart and their arms folded across their chest. When they felt ready, participants closed their eyes and informed the researcher. The researcher then started recording data using PASCO Capstone v2.5 at 100 Hz for 30 s. COP sway was assessed as the total path (mm) of displacement of the COP in the medio-lateral and anterior-posterior planes.

### 2.10. Timed Up-and-Go

The TUG test is used as a measure of functional mobility, gait, and falls risk [28]. Participants sat in a chair with their back against the chair back. On the start command, participants rose from their chair, walked 3 metres, turned at a cone, walked back to the chair, and sat down. Timing began at the instruction to start, and ended when the patient was seated.

### 2.11. Five Chair Stands

The five chair stands test is used as a measure of functional lower extremity strength, balance, and falls risk in older adults [29,30,31]. Participants sit in a chair with their back against the chair back. On the start command, participants rise from the chair, and sit down for a total of five repetitions. Timing began at the instruction to start and ended when the patient had returned to being seated for the fifth time.

### 2.12. Data Analysis

Data are presented as mean ± standard deviation unless specified otherwise. One-way ANOVAs with Tukey post hoc analysis were used to assess for between-group differences in participant characteristics, 24 h dietary intake, and MIVC values during the familiarisation visit. Exploratory analysis was conducted for all other outcome measures and is presented in the Supplementary Materials.

## 3. Results

### 3.1. Participant Characteristics

Eleven older adults (*n* = 6 male; *n* = 5 female) were successfully recruited to this study. One female experienced knee pain following the familiarisation visit and withdrew from the study. Therefore, data are presented for ten participants (*n* = 6 male; *n* = 4 female). The retention rate was 91%. The minimum sample size was not achieved. A summary of baseline characteristics can be found in Table 2. No significant differences were present between groups across all participant characteristics.

### 3.2. Outcome Measures

This study was underpowered for statistical analysis. Participants reported full adherence to the nutritional intervention. A summary of the data can be found in Table 3.

## 4. Discussion

Recovery from resistance exercise in older adults is an emerging and important consideration in exercise sciences, but the literature surrounding the topic is limited and inconsistent [4]. This was the first study that attempted to investigate the effectiveness of a nutritional intervention for improving exercise recovery, or attenuating exercise-induced muscle damage, in older adults following RE. Due to challenges with recruitment, the study was not adequately powered to detect group differences; hence, all results should be interpreted with caution, and no conclusions should be drawn as to the effectiveness of whole milk as an exercise recovery supplement. Despite this, this study does provide important insights that could help to inform the design and delivery of future research in this area.

## 5. Challenges in Conducting Exercise Recovery Studies in Older Adults

### 5.1. Recruitment

As mentioned previously, this study encountered substantial recruitment issues, recruiting only 10 of the 36 participants needed for the required statistical power. This may be due to a variety of reasons, and all of them should be considered in any future research aiming to recruit older adults for similar studies in the United Kingdom. During the six-month recruitment period, only 11 older adults were recruited to the study. Therefore, not only was the recruitment window limited, but recruitment within this was difficult. It may be that specific elements of the study design deterred people from participation. For example, several individuals could not make the repeated study visits fit with their schedule, others did not want to drink cow’s milk, and some did not want to complete RE, or already participated in RE. The pre-exercise health screening also limited recruitment numbers as individuals could not participate without passing a PAR-Q+. Had recruitment continued for a longer period, statistical power may have been achieved. It cannot be said what effect COVID-19 had on older adults’ willingness or ability to participate in this study approximately twelve months after the last national lockdown, but other studies have reported that participation rates were similar to those seen pre-pandemic [32]. At present, it is unclear how these obstacles to recruitment could be overcome for such a study, given that these issues are integral to the study design. It is possible that similar studies may have to plan for a longer recruitment period in lieu of altering recruitment rate or work collaboratively with other research centres to reach a wider population of people in order to achieve sufficient statistical power.

### 5.2. Measurement of Physical Function in Older Adults

Within muscle damage studies of younger adults, it is expected that physical function will be decreased at 24–48 h after exercise and return to baseline within 96 h [33]. In older adults, this temporal pattern is not as well established within the literature due to differing exercise protocols and outcome measures [4]. Within current studies, it is estimated that muscular strength of the lower limbs could be decreased by 9–36% following resistance exercise [4]. However, this was not observed in the present study. It is possible that any decrements in physical functioning resulting from RE were masked by a learning effect in this population, potentially coupled with a lack of exercise-induced muscle damage, which will be discussed subsequently. Indeed, when assessing the results of the whole milk and skimmed milk groups performing MIVC, values were higher than baseline in all time points following exercise. It is possible that the older participants were still learning how to produce a maximal isometric contraction up to 72 h, and this resulted in an apparent lack of change in maximal isometric strength following the RE. Indeed, even withstanding a lack of muscle damage, it is probable that MIVC would fluctuate around baseline values due to random error rather than steadily increase, which is the case in these data, if there was no learning effect [34]. This may otherwise be referred to as systematic bias, and is common in variables relevant to physical functioning [35]

Additionally, TUG performance was improved at 48 and 72 h after exercise, compared with immediately post-exercise, which could indicate a learning effect or substantial random error. The expectation would be for individuals to take more time to complete the test if muscle damage were present, as previous studies have shown a 2–18% [20,36] peak increase in time to complete the test. Baseline values of TUG speed (~8 s) in the present study are not likely to be a reason for this difference, with the previous studies reporting similar baseline speeds of 6 to 11 s [20,36], and the normative value for TUG being 9.2 s in 70–79-year-olds [37]. Generally, the TUG test has a high repeatability in older adults (*r* = 0.90–0.97) [38,39], but it is unclear from the published studies if participants had already undergone substantial familiarisation to obtain these values. These studies also do not appear to investigate temporal effects; for example, one study evidenced a high reliability for the TUG test (*r* = 0.960), but the mean times to complete the TUG test reduced consistently each day for five days, resulting in a difference between day one and day five of 0.82 s (~8%) [39]. If this variation is also present in our study, which is likely due to the small sample size, it may explain the decrease (~0.7 s) in time to complete the TUG test from post-exercise to 48 and 72 h, whilst not discrediting the previous studies that have shown an effect of muscle damage [20,36].

### 5.3. Familiarisation

Learning effects are common when assessing maximal strength. As previously discussed, they are an outcome of systematic bias [34] and are moderated through familiarisation before experimental visits [40,41]. In younger adults, repeated repetitions of MIVC are generally recommended before a plateau is achieved in peak MIVC values [42], but the number of familiarisation visits required for older adults is unclear [41,43]. Previously, a high short-term reliability across repeated trials has been demonstrated in leg extensor power of older adults (mean change 1.2–4.8% from trial 1 to trial 2) [44]. Likewise, when assessing bilateral concentric knee extensor 1-RM strength, untrained older adults may need up to nine testing sessions to achieve absolute consistency of this measure [45]. It has also been recommended that older adults may need 4–5 familiarisation attempts to achieve a stable baseline measure in the TUG test [46]. However, as far as we are aware, there is no comparable study for isometric strength reliability. If additional visits are required for familiarisation of MIVC measures in older adults, the practicability of this should be examined given that recruitment for this study was already difficult due to participant burden. Indeed, the addition of extra familiarisation visits would make future studies more methodologically rigorous but could discourage participation further.

### 5.4. Selection of Outcome Measures

Population-specific outcome measures are essential to ensure that research findings are relevant and applicable. This study was one of the first to attempt to use a number of outcomes that have been shown to be important for older people, including the TUG test, five chair stands, and COP sway, to investigate exercise recovery in older adults [4]. Previously, only two studies have reported on the effects of RE on TUG performance [20,36], two studies have reported postural stability (COP sway) [2,20], and one has reported five chair stands [47]. One study has investigated chair ascent and descent performance following RE in older adults, but this was not viable to measure in the current study [36]. Within this study, there was no apparent effect of RE on these parameters. It may be that the exercise protocol used in this study did not cause exercise-induced muscle damage. However, since these outcomes have not been validated for assessing muscle damage in this population, it cannot be said with any certainty whether there was no muscle damage, or whether these outcomes are unsuitable as a marker of exercise recovery. For example, although centre of pressure (CoP) sway was used in one study in older adults [20] who saw a 45% increase in CoP sway, it is not a well-used marker for exercise-induced muscle damage, and future studies may wish to validate its use for this purpose.

Similarly, muscle soreness is a regularly used marker of muscle damage in younger adults, which follows similar temporal patterns to the magnitude of muscle damage. Likewise, muscle soreness in older adults appears to peak at 24–48 h post-exercise and is largely recovered within three to five days [4]. However, in this study, the highest values for perceived muscle soreness were immediately post-exercise, and pre-exercise values were higher than 48 and 72 h post-exercise. Generally, individuals will experience no muscle soreness when they are rested, and soreness will peak 24–48 h after unaccustomed exercise [33]. The relatively high pre-exercise values indicate that, although visual analogue scales are widely used for assessing perceived muscle soreness, older adults in this study may have misunderstood what was meant by muscle soreness. This issue may be mitigated in two ways. Firstly, a brief explanation of delayed onset muscle soreness could be provided to individuals before the completion of any visual analogue scales, or alternatively via assessing the pressure pain threshold of participants, which would limit any variation caused by misunderstanding. The latter has not previously been used in older adults for muscle damage studies, and so may require some validation. More broadly, research should aim to identify any indirect markers of muscle damage that are both relevant and reliable in older adults that can be used in future studies.

### 5.5. Optimising Exercise Protocols

Intense or unaccustomed exercise is expected to cause exercise-induced muscle damage characterised by reduced muscular strength, power, and muscle soreness [33,48,49]. Exercise recovery studies observe these perturbations in exercise-induced muscle damage symptoms to indirectly assess levels of muscle damage and track recovery. In similar studies of older adults, maximal isometric knee extensor strength was decreased by 6–23%, and mild increases in muscle soreness were observed after RE [4]. The exercise protocols used in these previous studies include three sets to failure of 95% 5-RM of leg press and leg curl [36] and five sets of 15 reps of back squat at 75% 1-RM [50]. However, in this study, there was no discernible effect of the prescribed exercise on measures of physical function, muscle soreness, or perceived recovery. It is unclear if this is due to the RE causing no muscle damage and no fatigue, or an inability of the measurement tools and statistical power to detect such a change in this scenario.

Despite these limitations, it is also possible that the participants in this study did not experience exercise-induced muscle damage. The exercise protocol that was used (four sets of ten repetitions at 70% 1-RM of three lower limb exercises; leg press, knee extensions, and hamstring curls) should have been a significant stimulus to produce exercise-induced muscle damage in older adults [20,50], whilst attempting to replicate what would be considered a “typical” RE session for older adults rather than one that was intended to cause large amount of muscle damage. This exercise session was chosen to ensure that any findings were directly applicable to older adults, rather than a proof of concept. Another study previously used an RE session designed to replicate a “usual” training session in older adults, but they trained plantar flexors rather than knee extensors [20]. Hence, this protocol was not directly replicated from any previous studies, so it is hard to define the suitability of the exercise session for this study. Other studies [36,51] in older adults previously completed sets of exercises to failure to ensure that all participants reached volitional exhaustion and used this as a benchmark to standardise relative load across the population. This may be a good solution for further work but would require an estimation of 1-RM and would likely be subject to an individual’s motivation to exercise.

## 6. Considerations for Nutrition and Exercise Studies in Older Adults Arising from This Study

A summary of considerations for nutrition and exercise studies in older adults can be found in Table 4.

## 7. Conclusions

In conclusion, the methodological considerations for future studies investigating recovery from resistance exercise in older adults can be summarised as follows: (i) recruitment—consider ways in which the study design could be altered to aid recruitment or allow a longer recruitment period; (ii) learning effects and familiarisation—consider potential learning effects of outcome measures and adjust familiarisation accordingly (this may require additional research to be conducted first); (iii) identify, validate, and optimise protocols for outcome measures that are applicable for the specific population; (iv) adjust the exercise protocol according to the specific aims of the study (e.g., are you replicating a usual exercise bout or is the intent to cause large amounts of muscle damage?). These insights should be used to inform future research design to ensure that studies are methodologically robust and sufficiently powered to detect exercise-induced muscle damage in older adults.

## Figures and Tables

**Table 1:** Nutritional composition of drinks.

	Whole Milk (per 500 mL)	Skimmed Milk (per 500 mL)	Red Grape Juice (per 500 mL)
Energy (kcal)	325	185	320
Fat (g)	18	1.5	2.5
Carbohydrate (g)	23.5	24.5	114.5
Sugars (g)	23.5	24.5	105
Protein (g)	17	18	2.5
Salt (g)	0.05	0.5	0.4

**Table 2:** Participant characteristics.

Characteristics	Group		
	Whole Milk (*n* = 3)	Skimmed Milk (*n* = 4)	Control (*n* = 3)
Sex	M (*n* = 2); F (*n* = 1)	M (*n* = 2); F (*n* = 2)	M (*n* = 2); F (*n* = 1)
Age (y)	80 ± 5	74 ± 4	73 ± 2
Body mass (kg)	74.3 ± 6.0	71.8 ± 11.8	67.9 ± 8.3
Height (cm)	166.4 ± 3.2	165.4 ± 13.4	173.1 ± 11.0
Peak torque MIVC (Nm)	131 ± 78	127 ± 56	121 ± 33
Leg press 1-RM	62 ± 26	94 ± 9	72 ± 30
Knee extension 1-RM	80 ± 30	81 ± 18	83 ± 25
Hamstring curl 1-RM	75 ± 27	70 ± 20	78 ± 23
Exercise RPE (6–20)	13 ± 1	16 ± 3	15 ± 2
	24-Hour Dietary Intake		
Energy intake (kcal)	1435 ± 335	1861 ± 625	1748 ± 379
Protein (g)	62 ± 44	77 ± 19	69 ± 29
Carbohydrates (g)	162 ± 82	231 ± 86	195 ± 31
Fats (g)	44 ± 12	63 ± 41	56 ± 8

Data presented are means ± SD. Characteristics were measured during familiarisation, 24 h dietary intake was recorded for 24 h prior to the exercise bout. M: male; F: female; y: years; kg: kilograms; cm: centimetres; Nm: Newton metres; RAPA: rapid assessment of physical activity; 1-RM: one-repetition maximum; RPE: rating of perceived exertion; kcal: kilocalories; g: grams.

**Table 3:** Outcome measures.

Outcome	Time				
	Pre	Post	24 h	48 h	72 h
MIVC (Nm)	127 ± 51	136 ± 59	143 ± 57	145 ± 55	146 ± 59
Muscle Soreness (mm)	15 ± 11	25 ± 20	19 ± 17	10 ± 11	12 ± 9
COP Sway (mm)	1143 ± 754	1067 ± 561	1094 ± 621	1272 ± 873	1297 ± 841
TUG (s)	7.92 ± 1.56	7.89 ± 1.47	7.45 ± 1.55	7.23 ± 1.24	7.40 ± 1.38
Five Chair Stands (s)	11.37 ± 1.80	11.37 ± 1.93	11.29 ± 1.90	10.73 ± 1.91	10.54 ± 1.73

Data presented are means ± SD across all participants at each time point. TUG: timed up-and-go, COP: centre of pressure.

**Table 4:** Considerations for nutrition and exercise studies in older adults.

Methodological Consideration			
Recruitment	Learning Effects and Familiarisation	Population Specific Outcome Measures	Optimising Exercise Protocols
Consider recruiting individuals who already participate in resistance exercise. Consider the age limit for inclusion. Perform the study with appropriate clinical oversight to ensure older adults who are safe to exercise can be recruited. Consider, and plan for, a longer recruitment window if recruitment is expected to be difficult. Work collaboratively with other institutions to reach a greater audience.	Perform several familiarisation visits. OR choose outcome measures that have known high repeatability. Research is needed to confirm learning effects for muscle damage outcomes in older adults.	Research is needed to validate population specific outcome measures for exercise recovery in older adults. If outcome measures have been validated for other populations, consider if any adjustments need to be made (e.g., a more detailed explanation of muscle soreness may be required before assessment in older adults).	Consider the aims of your research when choosing exercise protocols. Do you intend to cause severe muscle damage or replicate usual training? Consider replicating an exercise protocol that has previously been used to cause muscle damage in your population.

## Data Availability

The raw data supporting the conclusions of this article will be made available by the authors on request.

## Supplementary Materials

The following supporting information can be downloaded at: https://www.mdpi.com/article/10.3390/geriatrics9050116/s1, Figure S1. Maximal isometric voluntary contraction values before and following a resistance exercise session presented as a percentage of pre-exercise values ± SD of the change. Figure S2. Perceived muscle soreness before and following a resistance exercise session (mean ± SD). Figure S3. Postural stability measured by centre of pressure sway before and following a resistance exercise session (mean ± SD). Figure S4. Time to complete the Timed Up-and-Go test before and following a resistance exercise session (mean ± SD). Figure S5. Time to complete five chair stands before and following a resistance exercise session (mean ± SD).
