# Supplementary material for: Challenges in Conducting Exercise Recovery Studies in Older Adults and Considerations for Future Research: Findings from a Nutritional Intervention Study
Source: Geriatrics (Basel). 2024 Sep 10;9(5):116. doi: 10.3390/geriatrics9050116 (PMC11417820; doi:10.3390/geriatrics9050116)
Supplement: Supplementary file 1 [file geriatrics-09-00116-s001.zip › geriatrics-3108395-supplementary.pdf]

Methodological considerations arising from a study investigating dietary interventions for recovery from resistance exercise in older adults – supplementary material

Exploratory Analysis

Maximal Isometric Voluntary Contraction

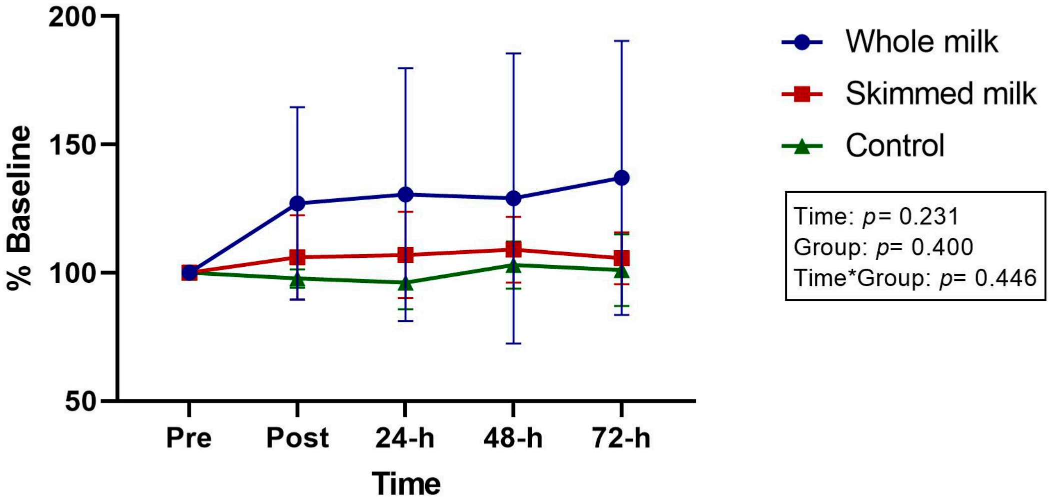

Figure s1. Maximal isometric voluntary contraction values before and following a resistance exercise session presented as a percentage of pre-exercise values ± SD of the change. Data were analysed using a mixed ANOVA. h: hours

Data for maximal isometric voluntary contractions (MIVC) of the knee extensors are presented as the average peak torque achieved over the four attempts at each time point (Figure 1). Absolute values for MIVC of the knee extensors were comparable between groups at baseline (Whole milk: 141.1 ± 87.7 N.m; Skimmed milk: 128.2 ± 66.9 N.m; Control: 139.8 ± 36.2 N.m; p= 0.961). Due to violations of sphericity, Greenhouse-Geisser corrections were applied when assessing percentage change from baseline. There was no significant effect of time on MIVC (p= 0.231), and no significant time*group interaction (p= 0.446).

Perceived Muscle Soreness

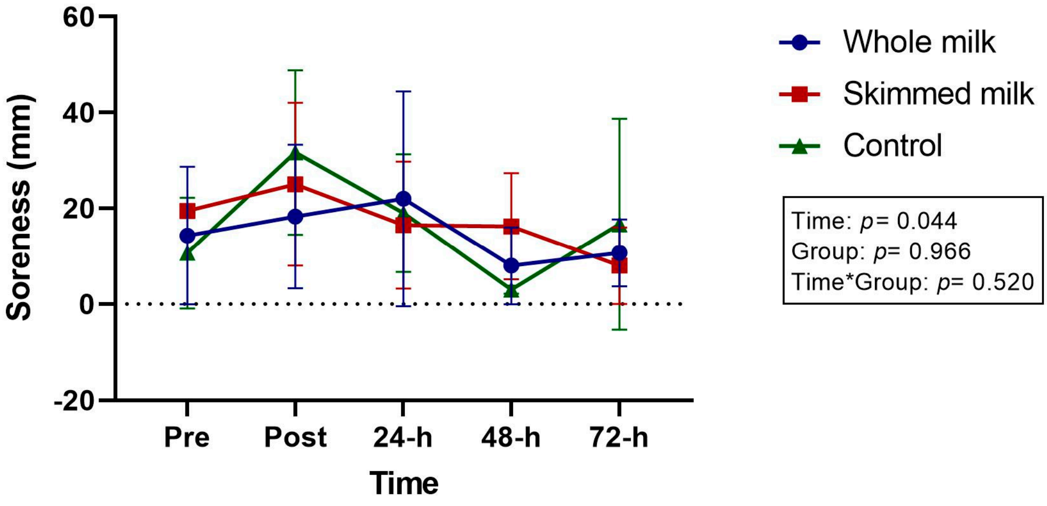

Figure s2. Perceived muscle soreness before and following a resistance exercise session (mean ± SD). Data were analysed using a mixed ANOVA with Bonferroni adjusted post hoc pairwise comparison. h: hours

Figure 2 shows groups means of muscle soreness throughout the study. There was no significant effect of group on perceived muscle soreness (p= 0.966). Mean baseline values for perceived muscle soreness were not significantly different between any groups (Whole milk: 14.3 ± 17.6 mm; Skimmed milk: 19.5 ± 1.7 mm; Control: 10.7 ± 14.2 mm), nor were they significant at any time point. Despite an overall significant effect of time F (4,28) = 423.733, p= 0.044, post-hoc tests revealed no significant differences between time-points. There was no significant time*group interaction (p= 0.520).

Postural Stability

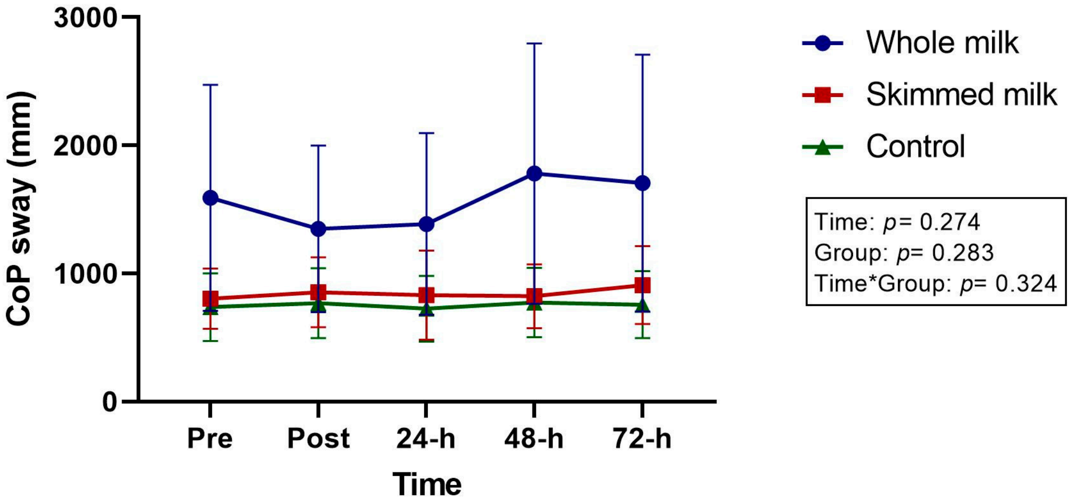

Figure s3. Postural stability measured by centre of pressure sway before and following a resistance exercise session (mean ± SD). Data were analysed using a mixed ANOVA. CoP: centre of pressure; h: hours

The mean of the three 30-second attempts at each time interval was calculated and taken as an individual value for analysis. These data are presented in Figure 3. There was no significant effect of group on centre-of-pressure (CoP) sway (*p*= 0.283). Greenhouse-Geisser corrections were applied to repeated measures analysis. CoP sway remained unchanged at each time interval (*p*= 0.274), and there was no time*group interaction (*p*= 0.324).

Timed Up-and-Go

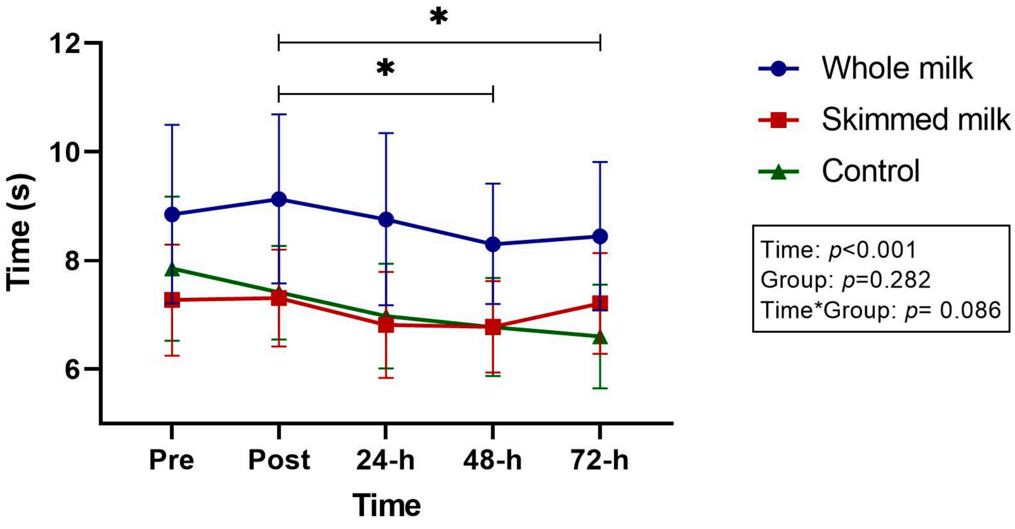

Figure s4. Time to complete the Timed Up-and-Go test before and following a resistance exercise session (mean ± SD). Data were analysed using a mixed ANOVA with Bonferroni adjusted post hoc pairwise comparison. h: hours

Mean times to complete the TUG test are displayed in Figure 4. There was a significant main effect of time on the time to complete the TUG test, F (4,28) = 9.178 (p< 0.001). Contrasts revealed that participants were quicker to complete the TUG test at 48-h (7.29 ± 0.36 s) and 72-h (7.42 ± 0.42 s) when compared with immediately post-exercise (7.95 ± 0.51 s, p< 0.05). There was no statistically significant difference between groups (p= 0.282), and no statistically significant time*group interaction p= 0.086).

Five Chair Stands

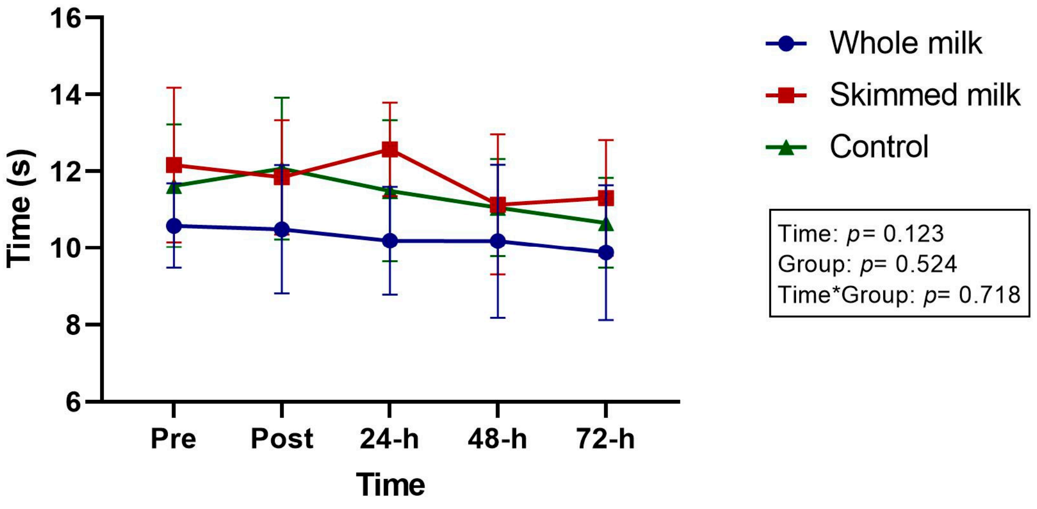

Figure s5. Time to complete five chair stands before and following a resistance exercise session (mean ± SD). Data were analysed using a mixed ANOVA. h: hours

Mean times to complete the five chair stands test are shown in Figure 5. Time to complete five chair stands was comparable between groups at all time points (*p*= 0.524) and did not significantly change as the study progressed (*p*= 0.123). There was no time*group interaction (*p*= 0.718).
